# Prevalence and predictors of false positive QTc prolongation by the automated measurement

**DOI:** 10.3389/fcvm.2024.1465264

**Published:** 2024-10-10

**Authors:** Wael Alqarawi, Marwah Allwaim

**Affiliations:** Department of Cardiac Sciences, College of Medicine, King Saud University, Riyadh, Saudi Arabia

**Keywords:** atrial fibrillation, QT measurement, tangent method, automated QT, Bazett's formula

## Abstract

**Background:**

Corrected QT (QTc) is an important electrocardiographic (ECG) interval. Physicians rely on automated QTc provided by ECG machines while the manual method is the recommended method. We sought to assess the prevalence and predictors of false positive QTc prolongation by the automated measurement.

**Methods and results:**

Consecutive ECGs were retrieved from the ECG database at King Khaled University Hospital. Manual QT was measured by a trained physician using the tangent method and was corrected for heart rate and QRS duration. Automated QTc measurement was recorded by the ECG machine. “Long QT (LQT)” was defined as QTc≥470 ms for males and ≥480 for females. False positive LQT was defined as LQT by automated QTc but not manual QTc. Pre-determined factors were included in a multivariate logistic regression to assess predictors of false positive LQT. A total of 567 ECGs were included in this study. Automated QTc was longer than manual QTc (440 ms [±35] vs. 417 ms [±35], respectively) which resulted in a high negative predictive value (NPV) (99%) and a low positive predictive value (PPV) (32%). Male gender and abnormal rhythm were found to be independently associated with false positive LQT (OR = 1.9 [95% 1.1–3.5], *p* = 0.03 and OR = 3.1 [95% 1.2–8.3], *p* = 0.02; respectively).

**Conclusion:**

Automated QTc measurement is unreliable for detecting long QT, necessitating manual verification and further research to enhance its accuracy.

## Introduction

QT prolongation can lead to serious ventricular arrhythmias. As a result, QT measurement is an essential component of electrocardiogram (ECG) interpretation and must be performed carefully (1). While ECG machines automatically measure QT and provide corrected QT (QTc) interval, manual QT measurement is the standard method recommended by expert consensus documents (1). However, there is increased reliance on automated QT measurement where major decisions such as medication interruptions and delays in initiating QT-prolonging medications are based on these measurements.

Multiple factors can affect the QTc measurement including heart rate, QRS duration, RR variability and T wave morphology. Methods to account for these factors have been proposed such as the use of Bazett's formula for heart rate correction and the tangent method to determine the end of the T wave. Automated QTc measurement adjust for some but not all of these factors. As such, one can understand why automated QTc can reveal a different measurement than manual QTc. Indeed, few previous studies examined the correlation between automated QTc and manual QTc and reported overestimation of the automated QTc (1, 2). However, these studies were performed in a selected group of patients. Moreover, no study provided predictors of discrepant measurements between automated and manual methods. As such, we sought to compare automated with manual QTc measurement in an unselected group of patients to describe the prevalence and predictors of false positive QTc prolongation.

## Methods

This was a retrospective observational study done at King Khaled University Hospital (KKUH).

### Data source

MUSE (GE Healthcare, Waukesha, Wisconsin) ECG database at KKUH was accessed. Consecutive ECGs were retrieved including in-patients and out-patients. All ECGs were performed using The Marquette^™^ (GE) 12SL automated ECG interpretation system.

### Variables

We collected basic demographic data (age and sex). In addition, we collected electrocardiographic data such as heart rate, rhythm, intervals (QRS and QT), T wave morphology, presence of bundle branch block (BBB), premature beats, and artifacts. “Irregular rhythm” was defined as atrial fibrillation, premature ventricular contraction (PVC) or premature atrial contraction (PAC). “Long QT (LQT)” was defined as QTc ≥470 ms for males and ≥480 for females (3). The diagnosis of LQT was considered to be “suppressed” if there was LQT by automated QTc with no mention of “prolonged QT” on the machine's interpretation of the ECG. False positive LQT was defined as LQT by automated QTc but not manual QTc.

### QT measurement

Automated QT measurement was recorded by the ECG machine. Manual QT was measured by a trained physician using the tangent method (4). Briefly, the point where the tangent of the steepest terminal limb of the T wave meets the isoelectric line was used to define the end of the T wave. U waves were excluded.Lead II or V5 were used for manual calculation unless other leads had longer QT duration. A random sample (10% of the whole sample size) was reviewed by an electrophysiologist and the correlation was reported. In the case of atrial fibrillation (AF), we averaged the QT over five beats and in the presence of PVS or PACs, the beats immediately following the premature beat was avoided (5). We excluded ECGs where we were unable to measure QT such as bigeminal rhythm.

### Qtc measurement

Automated QTc measurement was recorded by the ECG machine. Manual QT was corrected for heart rate using the Bazzet's formula as our goal was to compare automated to manual measurements and, as such, we consistently used the same method (6). In addition, in the presence of a QRD duratin ≥120 ms, we corrected QT using the following formula [QTc = QTc—(QRS—100 ms)] (7).

### Ethical consideration

This study also received Institutional Review Board approval from King Saud University College of Medicine.

### Statistical analysis

A formal, *a priori* calculation of sample size was carried out. We estimated the prevalence of false positive LQT to be 10% based on a pilot of 150 ECGs. Before data collection, we selected 6 factors to be tested for independent association with false positive LQT. These factors were chosen based on previous literature and biologic plausibility and included: sex, heart rate, rhythm, BBB, “irregular rhythm” and U waves. As such, a sample size of ≥550 ECGs was needed to allow for a precise estimate of the prevalence and enough power to test for proposed predictors.

Continuous data were reported as means (±SD) and categorical data as numbers (percentages). Student *T*-test, Chi Square test and Fisher's exact test were used when appropriate to analyze data. Sensitivity, specificity, positive and negative predictive value were calculated assuming manual QTc measurement to be the gold standard. We used multivariate logistic regression modeling to determine independent predictors of false positive LQTs. All pre-determined factors were included in the model. Correlation and agreement between readers were assessed with Pearson's correlation coefficient (r) and Kappa statistics, respectively. Analyses were performed using SAS (version 9.4, The SAS institute, USA) and *P* values of <0.05 were considered statistically significant.

## Results

### Overall characteristics

**Figure 1:**
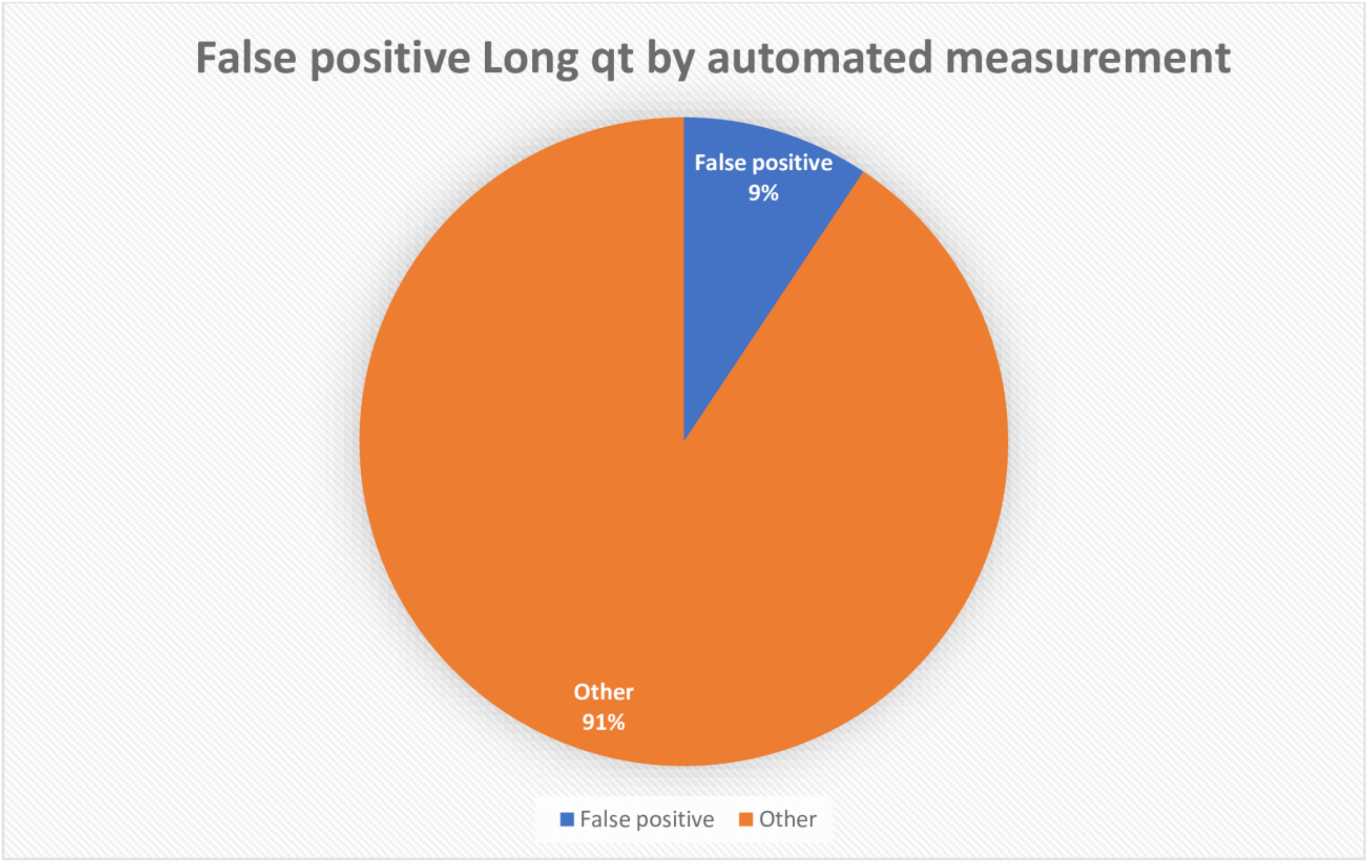
Prevalence of false positive long QT by automated QTc measurement.

A total of 567 ECGs were included in this study. The mean age was 52 (±18) years and 53% were females. Only 17 ECGs (13%) were done for patients under the age of 18.Most patients had sinus rhythm (95%) followed by AF (3%). Irregular rhythm was seen in 8.5% and 4% of all ECGs had BBB. On average, automated QTc was longer than manual QTc (440 ms [±35] vs. 417 ms [±35], respectively). The mean difference in QTc between automated and manual QTc was 32 (±72) ms and the mean difference in QT between automated and manual QTc was 18 (±17) ms. Table 1 summarizes the overall characteristics and Figure 1 shows the prevalence of false positive LQT.

**Table 1:** Overall characteristics.

Variable	Results (*N* = 567)
Age (y)	52 (18)
Heart rate (beats/min)	83 (19)
Sex (F)	52%
Rhythm	
– Sinus	95%
– Paced	0.2%
– Atrial fibrillation	3%
– Other	1.4%
Irregular rhythm	8.5%
Bundle branch block	4.4%
Artifact	16%
Premature atrial beats	1.76%
Premature ventricular beats	4%
U wave	0.7%

Data presented as means (SD) or numbers (percentages).

There was strong correlation between QT measurements performed by the 2 readers (*r* = 0.8, *P* = 0.02) and excellent agreement in detecting LQT (Kappa = 0.8, *P* < 0.03).

### Performance of automated QTc measurement

As shown in Table 2, automated QTc measurement was found to have a high negative predictive value (NPV) (99%) and a low positive predictive value (PPV) (32%).

**Table 2:** Sensitivity and specificity of automated QTc measurement.

	No long QT (manual)	Long QT (manual)
No long QT (automated)	485 (85%)	4 (0.7%)
Long QT (automated)	53 (9%)	25 (4.4%)

### Predictors of false positive QTc prolongation

Table 3 compares the characteristics of ECGs with false positive LQT to the rest of the cohort. False positive LQT were more likely to be done for females (20/53 [38%] vs. 278/514 [54%], *p* = 0.036) and less likely to have sinus rhythm (47/53 [88%] vs. 494/514 [96%], *p* = 0.031).

**Table 3:** False positive vs. others.

Variable	False positive (*n* = 53)	Other (*n* = 514)	*P*-value
Age (y)	59 (17)	51 (18)	0.51
Heart rate (beats/min)	86 (17.5)	82.5 (19)	0.15
Sex (M)	20 (53%)	278 (54%)	0.02
Rhythm			
– Sinus	47 (88%)	494 (96%)	<0.001
– Paced	1 (1.89%)	0 (0%)	
– Atrial fibrillation	2 (3.7%)	15 (3%)	
– Other	3 (5.6%)	5 (1%)	
Irregular rhythm	7 (13%)	41 (8%)	0.19
Bundle branch block	5 (9%)	20 (4%)	0.06
Artifact	8 (15%)	85 (16%)	0.72
Premature atrial contraction	1 (2%)	9 (1.75%)	0.91
Premature ventricular contraction	5 (9.4%)	19 (3.7%)	0.04
U wave	0 (0%)	4 (0.7%)	0.51

Data presented as means (SD) or numbers (percentages).

Male gender and abnormal rhythm were found to be independently associated with false positive LQT in the multivariate regression analysis (OR = 1.9 [95% 1.1–3.5], *p* = 0.03 and OR = 3.1 [95% 1.2–8.3], *p* = 0.02; respectively). Table 4 shows results of the multivariate regression analysis.

**Table 4:** Multivariate regression.

Variable	OR	95% CI	*P* value
Male gender	1.9	1.1–3.5	**0** **.** **03**
Abnormal rhythm	3.1	1.2–8.3	**0**.**02**
PVC	2.2	0.7–6.3	0.15
Bundle branch block	2.4	0.8–7.1	0.12
Heart rate	1.0	0.9–1.0	0.44
Irregular rhythm	4.7	0.7–31.5	0.12

Bold values indicate statistically significant.

### Long QT diagnosis suppression by the machine

Of the 53 ECGs with LQT, the diagnosis of LQT was suppressed in 39 ECGs (40/53, 75%). There were no differences in the characteristics of ECGs with or without diagnosis suppression including the prevalence of false positive LQT (74% vs. 69%, *p* = 0.72 respectively).

## Discussion

Our study found automated QTc measurement to overestimate QTc which resulted in a low PPV and high NPV in diagnosing LQT. Moreover, LQT diagnosis was suppressed by the ECG machine in a high proportion of ECGs but was not helpful in reducing false positive results. Lastly, we found male gender and abnormal rhythm to be independent predictors of false positive LQT.

Our study is in line with previous studies that revealed the overestimation of QTc by automated measurement (2, 8, 9). This is likely due the difference in measuring the QT interval in addition to differences in correction methods. Indeed, we found the mean difference between automated and manual QT (18 ms) to only account for about 50% of the mean difference in QTc (32 ms). Automated QT measurement superimposes all leads to define the end of T wave which often leads to a longer QT as compared to the tangent method (1). However, this by itself only partially explain the difference and the rest is likely related to QTc correction. While ECG machines correct for heart rate using Bazzet's method, they do not adjust for QRS duration and it's unclear how it treats irregular rhythms and U waves. Also, differences in QT measurement are exaggerated when corrected for abnormal heart rates, given that Bazzet's formula divide the QT by the square root of RR intervals in seconds. Although BBB was seen more frequently in ECGs with false positive LQT (9% vs. 4%, *p* = 0.06), it was not found to be an independent predictor of false LQT. It is possible that the overestimation in patients with BBB is not big enough to result in a significantly higher proportion of LQT. Whether larger sample size would reveal a statistically significant association with BBB remains to be examined in future studies.

Male gender and abnormal rhythm were found to be independent predictors of false positive LQT. T wave morphology is known to be affected by sex which is probably the reason behind this independent factor (10). It is difficult to explain why abnormal rhythm is an independent predictor for false positive LQT given that irregularity, BBB and heart rate were adjusted for in the multivariate analysis. The small numbers of each abnormal rhythm preclude any further analysis to mechanistically understand reasons behind the overestimation in each abnormal rhythm. Future studies can focus on certain rhythm types which can help elucidate differences related to automated QTc measurements.

Our findings have several clinical implications. First, it provides evidence supporting the recommendations to verify automated QTc measurements and it quantifies the potential overdiagnosis when that is not done. The American heart association's scientific statement specifically indicates that it is “essential to visually validate QT-interval prolongation reported by a computer algorithm” (1). Second, given the high NPV of automated measurement, non-experts who are not comfortable with manual measurements can use automated QTc as a screening tool to rule out LQT. Notably, only 4 ECGs (0.7%) had a normal QTc by automated measurement but LQT by manual measurement, however, none of these ECGs had severe LQT (i.e., QT >500 ms) and 2 of them had borderline QTc by automated measurement (475 and 477 ms). Nonetheless, in the presence of high suspicion or risks, one needs to verify automated QTc even when it reports a normal QTc. Last, no important clinical decisions related to QTc such as stopping medications, delaying important therapies or diagnosing congenital long QT syndrome should be made based on automated QTc measurement. The low PPV of automated QTc and the high prevalence of false positive LQT argue that potential harm could be seen if decisions are made based on automated QTc without verifications. Unfortunately, machine suppression of the LQT diagnosis was not helpful in reducing the rate of false positive results, despite a high proportion of suppression. From a research perspective, we hope our findings will ignite interest in understanding reasons behind QTc discrepancy and determinants of LQT suppression. Improvement in automated algorithm can render automated QTc more helpful, especially for non-experts.

To our knowledge, our study is first to compare the automated with manual QTc in an unselected group of patients. This is important because the ECG is one of the most common tests performed for all patients, so it was crucial avoid excluding any patients to minimize selection bias. However, our study has several limitations. First, we relied on the tangent method to manually measure QT, which is controversial. There is no one universally agreed upon way to manually measure QT. Nonetheless, the tangent method is the basis of QT measurement in many clinical studies related to QTc (3, 8, 11, 12). As such, clinical decisions based on these studies should use similar QT measurement methods. Moreover, the tangent method is a reliable and reproducible method as shown in our study. Second, only a small number of patients included were younger than 18 year-old and data on patient athletic status were not collected. The accuracy of Bazett's formula in athletes and young patients have been questioned. However, our goal was to compare automated to manual measurements and, as such, we consistently used the same method. Third, we did not collect outcome data. LQT is an ECG finding that is only important if it is associated with worse clinical outcomes. While numerous previous studies have shown an association between prolonged QTc and worse outcomes, it is unclear whether the magnitude of difference seen between automated QTc and manual measurement would lead to any clinically important outcomes. Nonetheless, the high prevalence of false positive LQT seen in our study would likely lead to inappropriate stoppage of medications, unnecessary delays in therapies and anxiety which are enough to render this difference a clinically important one. Notwithstanding that, future studies can collect actions based on automated QTc measurement to robustly quantify the effect of false positive LQT. Last, our results should not be generalized to all ECG machines as algorithms of measuring QT intervals might be different among different venders.

## Conclusion

We compared automated QTc measurement with manual QTc measurement using the tangent method and reported an average overestimation of 32 ms. Automated QTc had a high NPV but low PPV in diagnosing LQT. Physicians should be aware of this limitation of automated QTc and further research should elucidate reasons for this discrepancies to improve automated QTc. Until then, automated QTc should be verified by manual measurements.

## Data Availability

The raw data supporting the conclusions of this article will be made available by the authors, without undue reservation.
